# Two approaches, one goal: rethinking breastfeeding promotion in maternity hospitals

**DOI:** 10.1007/s00431-026-07557-3

**Published:** 2026-10-24

**Authors:** Riccardo Davanzo, Fabio Mosca, Massimo Agosti

**Affiliations:** 1 Nutrition Research Centre, University of Varese, Insubria, Italy; 2 Department of Clinical Sciences and Community Health, University of Milan, Dipartimento Di Eccellenza, 2023-2027 Milan, Italy; 3 Pediatric Department, Hospital ‘F. Del Ponte’, University of Insubria, Varese, Italy

**Keywords:** Breastfeeding, Maternity hospital, Hospital policy, Baby Friendly Hospital Initiative

## Abstract

We compare the Baby Friendly Hospital Initiative (BFHI), a leader in promoting breastfeeding in maternity hospitals, and the innovative Hospital Policy on Breastfeeding (HPB) Project funded and managed by the Italian Society of Neonatology. The HPB Project’s approach can be described as pragmatic in terms of strategy (free of charge for participating facilities), recruitment process (interprofessional rather than top-down), and management. Furthermore, while the HPB Project complies with EU regulations and restricts the prescription of formula to exclusively breastfed newborns discharged from maternity hospitals, it is not bound by strict adherence to the International Code of Marketing of Breast-milk Substitutes, which, although commendable, has so far prevented a wider adoption of the BFHI initiative. The HPB Project could play a supportive role alongside the BFHI initiative, emphasizing the common goal rather than the differences.

*Conclusion*: The HPB Project might serve as a model to be replicated internationally.

**Table:** 

**What is Known:** *• The implementation of the Baby Friendly Hospital Initiative (BFHI) has proven to promote breastfeeding in maternity hospitals (MHs).* *• In Italy, the BFHI has a slow progression with only 36 certified Baby Friendly Hospitals (BFHs).*	
**What is New:** *• The Hospital Policy on Breastfeeding (HPB) Project represents an effective, pragmatic approach to promote the initiation of breastfeeding in Italian MHs.* *• The HPB Project might serve as a model to be replicated internationally.*

**What is Known:**

*• The implementation of the Baby Friendly Hospital Initiative (BFHI) has proven to promote breastfeeding in maternity hospitals (MHs).*

*• In Italy, the BFHI has a slow progression with only 36 certified Baby Friendly Hospitals (BFHs).*

**What is New:**

*• The Hospital Policy on Breastfeeding (HPB) Project represents an effective, pragmatic approach to promote the initiation of breastfeeding in Italian MHs.*

*• The HPB Project might serve as a model to be replicated internationally.*

## Introduction

In the context of the multifactorial influences on breastfeeding, the role of maternity hospitals (MHs) is relevant. Healthcare professionals working in MHs are called to implement supportive perinatal, postnatal practices, remove barriers, and provide personalized, evidence-based care that respects each woman’s needs, preferences, and choices [1]. Given this premise, both the BFHI [1] and the HPB Project [2] make available an operational framework for a multi-component structured intervention in MHs aimed to facilitate breastfeeding and to optimize infant nutrition and maternal health.

## The Baby Friendly Hospital Initiative

UNICEF and the WHO launched the “Baby-Friendly Hospital Initiative” (BFHI) in 1991 to promote, support, and protect breastfeeding. Since then, although up-to-date and precise statistics are not available, more than 20,000 health facilities in over 150 countries have been designated “Baby-Friendly.” In addition to the fact that the BFHI has a significant effect on breastfeeding in the period immediately following birth, systematic reviews conducted by Fan [3] and Habte [4] show that infants born in BFHI-accredited hospitals are also more likely to be exclusively breastfed for the first 6 months of life.

However, the BFHI initiative has some limitations. First, Fair [5] assessed the overall reliability of the evidence as low, primarily due to the risk of selection bias and the cultural and organizational heterogeneity of MHs, which calls into question its generalizability. Second, since the BFHI initiative is a multifactorial program that includes the “10 Steps,” it is difficult to isolate the contribution of each component in determining the overall observed effect. Furthermore, the “10 Steps” may be implemented with varying intensity and fidelity across different contexts. This also refers to a specific component of the “10 Steps,” namely full adherence to the International Code of Marketing of Breast-milk Substitutes (“The Code”), adopted by the World Health Assembly of the World Health Organization (WHO) and UNICEF in 1981 to protect breastfeeding by putting an end to the unfair marketing of infant formula and baby bottles [6]. Although the impact of the Code is widely recognized [7], the significance of partial compliance with the Code—as required by the Italian Law—compared to full compliance remains largely unknown. Third, studies reporting on the effectiveness of the BFHI often limit their analysis to the initiation, exclusivity, and duration of breastfeeding. In contrast, “harder” clinical outcomes such as maternal and neonatal morbidity and maternal mental health are much less well documented. Fourth, obtaining a BFH certification can be challenging, costly, and difficult to maintain [8], which entails the need for demanding periodic re-certifications.

In summary, although the BFHI is evidence-based, it cannot be considered an indisputable benchmark, and other possible initiatives aimed at promoting breastfeeding in MHs should not be overlooked, provided they have proven to be effective.

## The Hospital Policy on Breastfeeding Project

In Italy, available data show low breastfeeding rates, particularly at the time of hospital discharge [9], while, unfortunately, only about 36 out of a total of more than 350 Italian hospitals have been accredited as “Baby-Friendly” [10] for various reasons [2]. In this context, and in line with a hospital’s goal of supporting the initiation of breastfeeding, the Italian Society of Neonatology launched the “Hospital Policy on Breastfeeding” (HPB) Project in early 2023. The HPB Project is a joint initiative of the Italian scientific societies involved in perinatal care, together with the national associations of midwives, nurses, and the family association Vivere Onlus [2].

A major contribution of the HPB Project was the demonstration of measurable effects on a relatively large scale in Italy. The final study [2] included 97 of the 104 maternity hospitals (MHs) initially enrolled, representing 25–30% of all Italian MHs. Increases were observed in exclusive breastfeeding at discharge, skin-to-skin contact, rooming-in, staff training, and the development of breastfeeding care protocols. Thus, an organizational intervention—led by a scientific society rather than UNICEF and implemented simultaneously as part of routine practice across multiple MHs—was associated with measurable improvements in care indicators.

The evocative title of the 2026 article “Not so ambitious, still effective” is almost a methodological statement. Although the HPB Project is clearly inspired by the international paradigm of the Baby-Friendly Hospital Initiative, it adopts a pragmatic approach, integrating key elements of breastfeeding promotion into the daily routine of Italian MHs. The HPB Project did not simply seek to “increase breastfeeding,” but also aimed to reduce variability in care practices among hospitals—an issue of great importance even in the healthcare systems of high-income countries.

The HPB Project was conducted independently of both the BFHI and the Italian BFHI Network, and it shares the same position as many European Pediatric Associations [11]—namely to limit the prescription of formula to exclusively breastfed newborns discharged from MHs—without, however, requiring healthcare providers to fully comply with the Code.

It is clear that the HPB Project has certain limitations. First, the 2026 article presents the results of an uncontrolled study, which means it is not possible to attribute the entire improvement to the intervention with certainty [12]. The absence of a control group implies that the change may have been influenced by temporal trends or other interventions that occurred simultaneously. Second, the study sample was selected with MHs in northern Italy overrepresented. Third, the magnitude of the effect was somehow modest, with an increase in exclusive breastfeeding from 67.3% to 71.0%, although over the course of only 18 months [2].

In summary, the HPB Project has demonstrated that scientific societies can mobilize midwives, nurses, pediatricians, neonatologists, obstetricians, and family associations to change organizational practices in MHs. The HPB is not “a new model alternative to the BFHI” (Table 1), but rather an example of large-scale implementation of hospital policies supporting breastfeeding, characterized by a sense of realism, measurable effects, and significant potential for transferability. This is precisely what would make the project innovative from an international perspective, particularly for MHs that are not yet ready to tackle the challenges of the BFHI, serving as a useful support for the BFHI and, hopefully, a source of inspiration.

**Table 1:** Key features of the HPB Project compared with the BFHI

	HPB Project	BFHI
Goal	Promotion of breastfeeding upon discharge from the MH	Promotion of breastfeeding upon discharge from the MH
Promoting institution	Scientific Societies (SIN, SIP, SINUPE, AOGOI, SIGO, SININF, SIPINF), Nurses and Midwife Boards (FNOPO, FNOPI), family association (VIVERE Onlus)	UNICEF & BFHI Network
Project management	By the Italian Society of Neonatology	By UNICEF
Multi-component intervention	• Hospital breastfeeding policy, ad hoc working group, staff training, monitoring of breastfeeding rates, skin-to-skin contact, extended rooming-in (at least 20 h out of 24), breastfeeding protocols (coordinated by the SIN) • Partial compliance with “The Code” required	• 10 steps • Full compliance with “The Code” required
Enrolment strategy	• Invitation from the Italian promoting organization to directors of neonatal, pediatric, and obstetrics departments at all Italian MHs • Subsequent submission of an application by the hospital director • Moderately selective settings	• On a voluntary basis or following an agreement between UNICEF and the Regional Department of Health • Subsequent submission of an application to UNICEF by the hospital director • Highly selective settings
Registration fee	None; the project is funded by the Italian Society of Neonatology	In 2023, 5.500–17.000 € depending on the size of the healthcare facility (information available at: https://www.unicef.it/media/contributi-ricevuti-nel-2023-da-enti-pubblici-in-italia/)
Effectiveness	Lower • Uncontrolled “before-after” design • Long-term breastfeeding outcomes not assessed	Higher • Nevertheless evidence from a large RCT (PROBIT) has limited applicability [13] • Improvement in long-term duration of breastfeeding
Targets	Lower targets for exclusive breastfeeding rates at hospital discharge	Higher targets for exclusive breastfeeding rates at hospital discharge
Accreditation	• Certification: “Maternity Hospital for Breastfeeding” • Remote mentoring by SIN • No on-site visits • Self-assessment (potentially less accurate)	• Certification: “Baby Friendly Hospital” • Remote mentoring by UNICEF • On-site visits • External evaluation by UNICEF staff • Difficulty in maintaining high standards (need for ongoing reevaluations)
Innovation	• Reduced resource requirement • Pragmatic approach • Potential support to the BFHI	• Well-established initiative • A certain degree of dogmatism (no compromise regarding full compliance with the Code)
Public health relevance	Probably high	High
Implementation value	• Moderate • Greater potential to recruit a larger number of MHs	• High • Risk of excluding MHs with worthwhile but less ambitious targets

## Data Availability

No datasets were generated or analysed during the current study.

## Abbreviations

BFHI: Baby Friendly Hospital Initiative
HPB: Hospital Policy on Breastfeeding
MH: Maternity Hospital
UNICEF: United Nations International Children’s Fund
WHO: World Health Organization
